# N-Acetyl-L-glutamate Kinase of *Chlamydomonas reinhardtii*: In Vivo Regulation by PII Protein and Beyond

**DOI:** 10.3390/ijms241612873

**Published:** 2023-08-17

**Authors:** Vitalina Vlasova, Tatiana Lapina, Vladislav Statinov, Elena Ermilova

**Affiliations:** Biological Faculty, Saint-Petersburg State University, 199034 Saint-Petersburg, Russia; derkachvita99@gmail.com (V.V.); t.lapina@spbu.ru (T.L.); st067882@student.spbu.ru (V.S.)

**Keywords:** N-Acetyl-L-glutamate kinase, arginine biosynthesis, PII- signal transduction protein, green algae

## Abstract

N-Acetyl-L-glutamate kinase (NAGK) catalyzes the rate-limiting step in the ornithine/arginine biosynthesis pathway in eukaryotic and bacterial oxygenic phototrophs. NAGK is the most highly conserved target of the PII signal transduction protein in Cyanobacteria and Archaeplastida (red algae and Chlorophyta). However, there is still much to be learned about how NAGK is regulated in vivo. The use of unicellular green alga *Chlamydomonas reinhardtii* as a model system has already been instrumental in identifying several key regulation mechanisms that control nitrogen (N) metabolism. With a combination of molecular-genetic and biochemical approaches, we show the existence of the complex CrNAGK control at the transcriptional level, which is dependent on N source and N availability. In growing cells, CrNAGK requires CrPII to properly sense the feedback inhibitor arginine. Moreover, we provide primary evidence that CrPII is only partly responsible for regulating CrNAGK activity to adapt to changing nutritional conditions. Collectively, our results suggest that in vivo CrNAGK is tuned at the transcriptional and post-translational levels, and CrPII and additional as yet unknown factor(s) are integral parts of this regulation.

## 1. Introduction

Arginine and the regulation of its metabolism are of great interest in plant biology because this essential amino acid for protein synthesis is also a precursor in the formation of polyamines and nitric oxide, which play critical roles in plant development and adaptation to stresses [1,2,3,4]. The rate-limiting step in the ornithine/arginine (Arg) biosynthesis pathway is catalyzed by N-Acetyl-L-glutamate kinase (NAGK), which phosphorylates N-Acetyl-L-glutamate to N-Acetyl-L-glutamyl-phosphate [1]. The enzyme activity is negatively regulated by Arg feedback inhibition in Cyanobacteria and Archaeplastida (red algae and Chlorophyta) [5,6,7,8,9,10]. Through complex formation with protein PII, NAGK is relieved from inhibition by Arg [5,9,10,11,12], leading to enhanced activity.

PII proteins are among the most highly conserved and widely distributed signal transduction proteins known in all domains of life [11,12,13,14,15]. A phylogenetic analysis of PII homologs in the eukaryotic domain indicated their inheritance from a cyanobacterial endosymbiont, implying their restriction to Archaeplastida [16]. In contrast to Cyanobacteria and red algae, in green algae and land plants, NAGK activity is controlled by the cellular glutamine (Gln) levels via glutamine-dependent PII-NAGK complex formation, which leads to increased enzyme activity [9,10,17]. Gln sensing as the primary product of nitrogen (N) assimilation indicates the specialization of PII from green algae to respond to the cellular N status.

Among the green algae, *Chlamydomonas reinhardtii* (*Chlamydomonas* hereinafter) has become a good model system for revealing important facts about the regulation of N metabolism and has provided important insights for agricultural plants [18,19,20,21]. Progress has also been made in the identification of the genes and proteins of Arg biosynthesis [22,23,24,25]. According to our data, *Chlamydomonas* shares with higher plants and other green algae the capability of controlling via PII the activity of NAGK in vitro in a Gln-dependent manner [10]. Interestingly, in this alga, PII levels are controlled by the nitrogen source [26].

In Cyanobacteria, the PII protein is phosphorylated at a seryl residue (S49) located on the large surface-exposed T-loop [27]. The different NAGK activity observed in cyanobacterial cells results from the different PII phosphorylation states [12]. In green algae, NAGK research has mainly focused on PII-dependent regulation in vitro [9,10,13]. Despite their role in expanding our knowledge of the structure and properties of NAGK, the in vitro protein systems have their limitations, particularly, their failure to fully recapitulate the native cellular environment. The apparent gap in the information on NAGK regulation in vivo prompted us to investigate this enzyme activity and expression in *Chlamydomonas* cells grown in various N sources and under N deprivation.

The present study is the first to address the multiple control of NAGK in vivo, where the signal protein PII is only an integral part of the regulatory network.

## 2. Results

### 2.1. CrNAKG Expression Is Dependent on Nitrogen Source and Growth Phase

**Figure 1:**
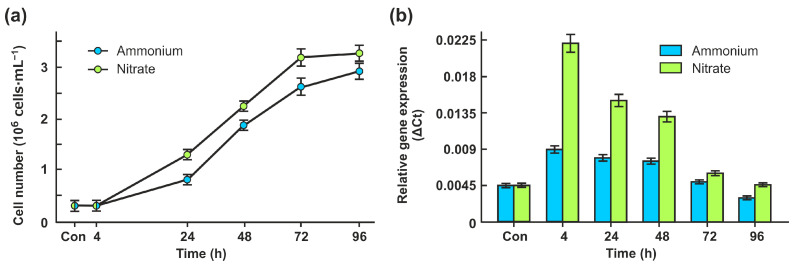
Effects of ammonium and nitrate on cell growth and relative Cr*NAGK1* gene expression. (**a**) The growth curves were analyzed in the presence of 7.5 mM NH_4_Cl or 4 mM KNO_3_. Values are means ± SE of three independent experiments; (**b**) Time course of the Cr*NAGK1* transcripts accumulation during growth of cells in ammonium- or nitrate-containing medium. Values are means ± SE of three biological replicates and three technical replicates and are given as expression level relative to a housekeeping gene *RACK1*.

We chose the wild type 6145c for experiments because it is an arginine prototroph and can utilize ammonium, nitrate, or nitrite as nitrogen sources [28]. In the first type of analysis, cell growth was compared on two nitrogen sources, ammonium and nitrate (Figure 1a). Although *Chlamydomonas* grew well on both media, ammonium supplementation resulted in slightly slower growth and lower final yields.

As shown in Figure 1b, the relative expression level of Cr*NAGK1* was higher in the nitrate-grown cells than in the ammonium-grown cells. Moreover, nitrate led to an approximately 5-fold increase in Cr*NAGK1* transcript abundance after 4 h (lag phase) and up to approximately 3.3-2.5-fold after 24 h and 48 h (early- and mid-log phases) of acclimation to nitrate. Interestingly, the accumulation of Cr*NAGK1* decreased again to the control level after 72 h (late-log phase). In the presence of ammonium, the expression levels of this gene in the lag, early- and mid-log phases were higher by about 2–1.7 times than in the late-log and stationary phases of growth (Figure 1b). Thus, Cr*NAGK1* transcription was influenced by the growth phases and N source.

### 2.2. CrNAKG Activity Is Dependent on Nitrogen Source

There is evidence that the activity of cyanobacterial NAGK is dependent on the nitrogen source [29]. We wanted to find out whether the CrNAGK activity also depends on which external nitrogen source was provided.

**Figure 2:**
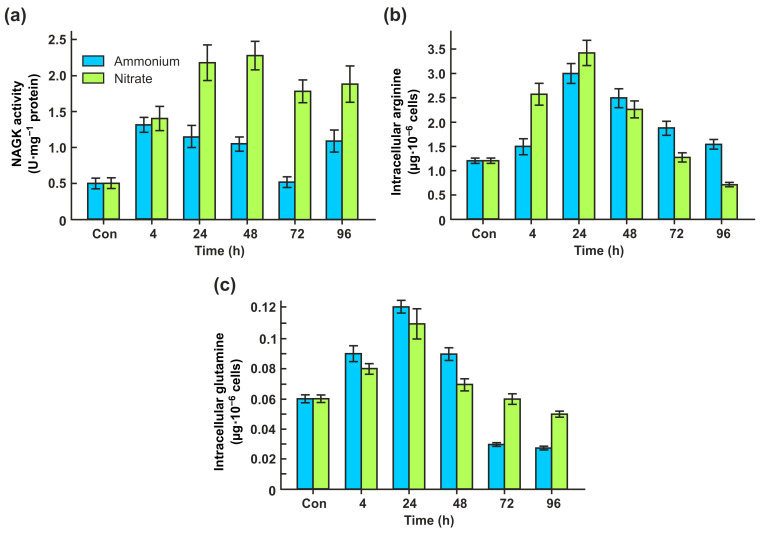
Effects of ammonium and nitrate on the specific activity of CrNAGK and the total free content of Arg and Gln. (**a**) Time course of the CrNAGK activity during growth of cells in ammonium- or nitrate-containing medium. Cells were grown as described in Figure 1a; (**b**) Relationship between nitrogen source and intracellular Arg content; (**c**) Relationship between nitrogen source and intracellular Gln content. In (**b**,**c**), the content of amino acids is expressed in µg per10^6^ cells. Values are means ± SE of three biological replicates.

In the presence of ammonium, the levels of CrNAGK activity increased at the lag phase (4 h) to a peak level of 1.32 U mg^−1^ protein and then declined slightly as the culture entered the early logarithmic (log) phase of growth, reaching its lowest level during the late log phase. (Figure 2a). Interestingly, a significant level of enzyme activity (1.1 U mg^−1^ protein) was detectable in the stationary phase.

When cells were transferred to a nitrate-containing medium, the CrNAG kinase activity was also increased after 4 h but reached a maximum within the early- and mid-log phases of growth (24 h and 48 h of incubation) (Figure 2a). During further growth in nitrate-containing medium, the enzyme activity decreased again to approximately 3.6 times higher than the control level. It appears likely that the regulation of CrNAGK expression in cells supplemented with ammonium or nitrate is not the only level of enzyme control, especially in the stationary phase of growth.

*Chlamydomonas* NAGK has been shown to be an arginine-sensitive enzyme [10]. In vitro, the activity of arginine-sensitive NAGKs is negatively regulated by arginine feedback inhibition [11,30,31,32]. To assess the overall impact of arginine on the CrNAGK activity in vivo, we quantified the intracellular free arginine content in cells grown on ammonium and nitrate as nitrogen sources. This amino acid content increased after 4 h and reached a maximum in the early-log phase of cell growth in both media (Figure 2b). During further growth, the intracellular arginine concentrations decreased. The observed enhanced level of internal arginine in the lag, early- and mid-lag phases correlated with a high level of CrNAGK activity in these phases (Figure 2a) with *r* of 0.50 and 0.59 for ammonium and nitrate, respectively. This indicates a relief of this enzyme from inhibition by arginine. In addition, the levels of Gln as an amino acid that controls CrNAGK activity [10] showed the same trend as Arg (Figure 2c). Notably, CrPII is also induced by nitrate [26]. Collectively, these data suggest that PII might be involved in the N-dependent control of enzyme activity.

### 2.3. Nitrite Promotes CrNAGK Activity

**Figure 3:**
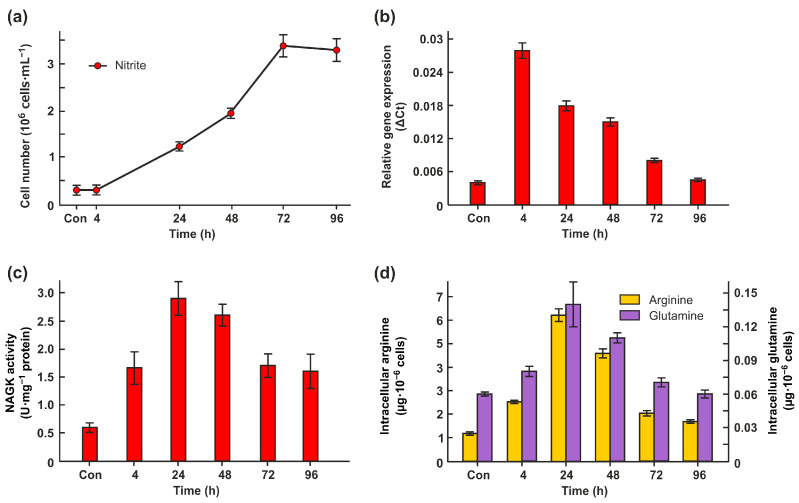
Effects of nitrite on cell growth, CrNAGK expression and activity, and the total free content of Arg and Gln. (**a**) The growth curve was analyzed in the presence of 10 mM KNO_2_. Values are means ± SE of three independent experiments; (**b**) Time course of the Cr*NAGK1* transcripts accumulation during growth of cells in nitrite-containing medium. Values are means ± SE of three biological replicates and three technical replicates and are given as expression level relative to a housekeeping gene *RACK1*; (**c**) Time course of the CrNAGK activity during growth of cells in nitrite-containing medium; (**d**) Relationship between nitrogen source and intracellular Arg and Gln content. The concentration of amino acids is expressed in µg per10^6^ cells. Values are means ± SE of three biological replicates.

To further explore the relationship between NAGK regulation and N sources, the alga was incubated in 10 mM nitrite. Cells of strain 6145c growing in nitrite-supplemented medium did not show significant differences in their growth characteristics compared with cells grown on ammonium (Figure 1a and Figure 3a).

In nitrite, cells exhibited the same patterns of *CrNAGK1* gene expression and enzyme activity as cells grown in nitrate (Figure 3b,c). The highest levels of cellular Arg and Gln were obtained in cells grown in the early- and mid-log phases (Figure 3d). Therefore, PII might dampen arginine feedback inhibition under these conditions.

### 2.4. Underexpression of CrPII Decreases the Activity of CrNAGK but Not the CrNAGK1 Transcript Level

**Figure 4:**
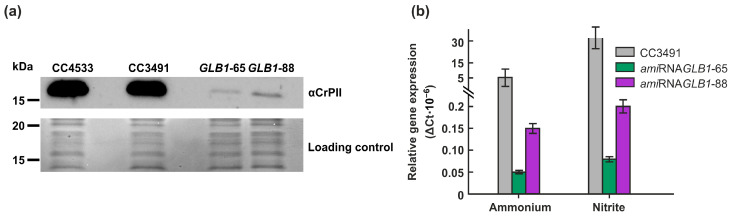
Characterization of *ami*RNA-*GLB1* strains. (**a**) CrPII abundance in wild-type strains (CC4533 and CC3491), *ami*RNA*GLB1*-65, and *ami*RNA*GLB1*-88. Protein levels were analyzed by Western blotting. Each line corresponds to 40 μg of soluble proteins extracted from samples taken from cultures incubated in nitrite-containing medium for 24 h. *GLB1*-66 and *GLB1*-88 indicate *ami*RNA*GLB1*-65 and *ami*RNA*GLB1*-88, respectively; (**b**) RT-qPCR analysis of *CrGLB1* transcript levels. Relative expression levels were normalized with the gene expression of *RACK1* and calculated using ΔCT. Samples were analyzed from cultures incubated in ammonium- or nitrite-containing medium for 24 h. Values are means ± SE of three biological replicates and three technical replicates.

To clarify the role of the CrPII protein in CrNAGK regulation in vivo, we generated Cr*GLB1*-underexpressing transformants as described previously [33]. Two *ami*RNA*GLB1* strains were selected for further research. Western blotting revealed that the CrPII protein levels in the *ami*RNA*GLB1*-65 and *ami*RNA*GLB1*-88 strains were no higher than 5% of those in the parental cells (Figure 4a). To test if the phenotype was a result of decreased transcript accumulation, RNA was extracted from CC3491, *ami*RNA*GLB1*-65, and *ami*RNA*GLB1*-88 cells, and analyzed by quantitative real-time PCR. The results confirmed a significant knockdown of Cr*GLB1* mRNA in both transformants (Figure 4b).

The strain CC3491 was unable to grow on nitrate. However, this strain does exhibit growth on ammonium and nitrite as nitrogen sources. Notable, the downregulation of CrPII did not affect the growth of *ami*RNA*GLB1* cells (Figure S1).

**Figure 5:**
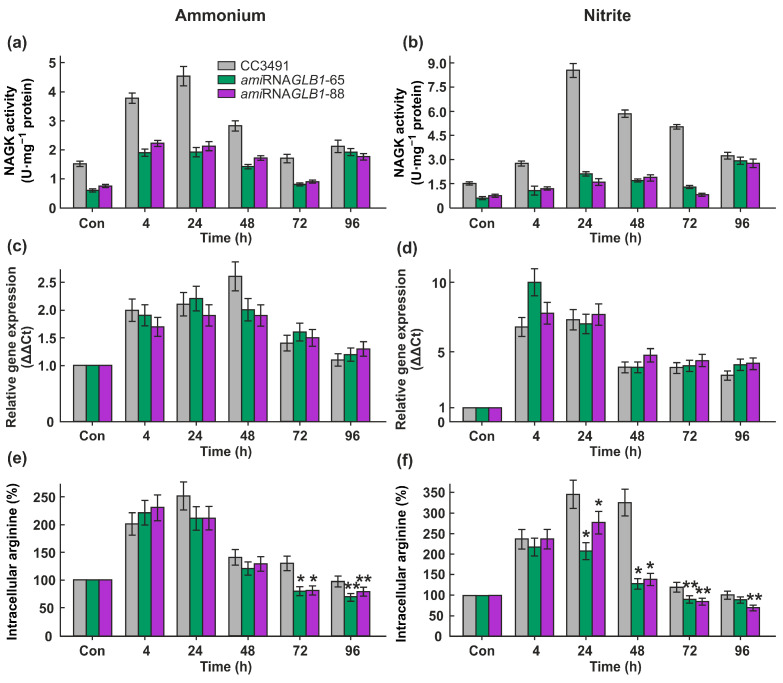
Effects of ammonium and nitrite on CrNAGK expression and activity, and the total free content of Arg in CC3491 and *GLB1*-knockdown strains. (**a**,**b**) Time course of the CrNAGK activity during growth of cells in ammonium- or nitrite-containing medium; (**c**,**d**) Time course of the Cr*NAGK1* transcripts accumulation during growth of cells in ammonium- or nitrite-containing medium. Values are means ± SE of three biological replicates and three technical replicates and are given as expression level relative to a housekeeping gene *RACK1*; (**e**,**f**) Relationship between nitrogen source and intracellular Arg content. Intracellular Arg concentration at 0 h in each strain is considered as control (set to 100%). Values are means ± SE of three biological replicates. * and ** denote significant differences between parental strain and Cr*GLB1*-underexpressing transformants according to the Student *t*-test (*p*-value < 0.01 or <0.05, respectively).

As shown in Figure 5a, the downregulation of Cr*GLB1* led to a decrease in CrNAGK activity both in ammonium-grown and nitrite-grown cells in the lag and log phases. Unexpectedly, there was no obvious difference in enzyme activity between the parent strain and Cr*GLB1*-underexpressing transformants during the stationary phase of growth (Figure 5a,b). However, the expression levels of the Cr*NAGK1* gene were similar to those of the parental strain (Figure 5b).

At the same time, lower enzyme activity correlated with a lower level of intracellular arginine in *GLB1*-knockdown cells compared with the parental strain in the late-log and stationary phases, and the log phase and in ammonium- and nitrite-containing medium, respectively (Figure 5c).

### 2.5. CrNAGK Activity Is Changed in N-Starved Cells

**Figure 6:**
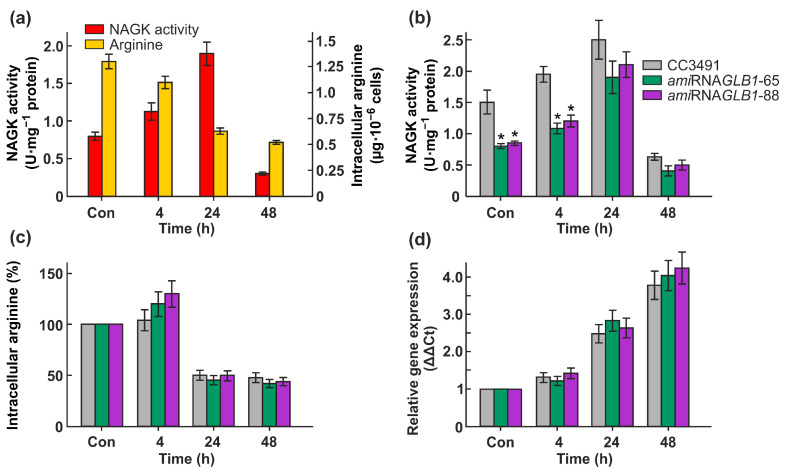
Effects of N deprivation on CrNAGK expression and activity, and the total free content of Arg in wild types and *GLB1*-knockdown strains. (**a**) CrNAGK activity and intracellular Arg content in 6145c strain during incubation in N-free medium; (**b**) Time course of the CrNAGK activity in parental strain and *ami*RNA*GLB1* strains during incubation in N-free medium. * denotes significant differences between parental strain and Cr*GLB1*-underexpressing transformants according to the Student *t*-test (*p*-value < 0.01 or <0.05, respectively, (**c**) Intracellular Arg content in parental strain and *ami*RNA*GLB1* strains during incubation in N-free medium. Arg concentration in each strain in N-replete medium is considered as control (set to 100%). Values are means ± SE of three biological replicates; (**d**) Time course of the Cr*NAGK1* transcripts accumulation in parental strain and *ami*RNA*GLB1* strains during incubation in N-free medium. Values are means ± SE of three biological replicates and three technical replicates and are given as expression level relative to a housekeeping gene *RACK1*.

It was previously shown that both the Cr*GLB1* gene and the Cr*NAGK1* gene are inducible under N-deprivation conditions [22,25]. Since nothing is known about the CrNAGK activity in N-deplete cells, we measured it in strain 6145c. The transfer of the cells from growth medium into N-free medium led to an increase in NAGK activity of approximately 1.4-fold after 4 h and of up to approximately 2.4-fold after 24 h of acclimation to N limitation (Figure 6a). However, after 48 h, the activity of CrNAGK decreased sharply to the level of 0.3 U mg^−1^ protein. The observed Arg level in the cells after 48 h of N deprivation was also the lowest (Figure 6a).

To test whether CrPII controls CrNAGK activity in N-starved cells, we compared the enzyme activity in the parental strain and Cr*GLB1*-knockdown transformants. When CC3491 cells are submitted to N deprivation for 4 h or 24 h, this is accompanied by an increase in CrNAGK activity (Figure 6b). The observed enzyme activity of the transformants following the elimination of N from the medium for 4 h was approximately 1.8 times lower than that of the WT. It is interesting to note that after 24 h or 48 h of incubation in N-free medium, the CrNAGK activity of *ami*RNA*GLB1*-65 and *ami*RNA*GLB1*-88 cells was very similar to that of parental cells (Figure 6b), suggesting the independence of NAGK regulation from CrPII under these conditions. After prolonged N-starvation (24 h and 48 h), the observed Arg levels in all strains were about 50–40% of that of N-replete cells (Figure 6c).

Next, we asked whether the changes in CrNAGK activity in N-starved cells are accompanied by changes in the transcript levels. The expression levels of the gene of interest were upregulated by N depletion in both the CC3491 and *ami*RNA-*GLB1* strains (Figure 6d). Importantly, the induction of gene *CrNAGK1* was not reduced in the Cr*GLB1*-knockdown transformants. These results indicate a role of transcriptional regulation of the *CrNAGK1* gene in increasing enzyme activity after 4 h and 24 h of N-starvation. In contrast, despite the increase in the amounts of CrN*AGK1* transcripts during further incubation in N-free medium (Figure 6d), the enzyme activity significantly decreased (Figure 6b), hinting at potential additional regulator(s) of NAGK during *Chlamydomonas* acclimation to N-starvation.

## 3. Discussion

NAGK is the most highly conserved target of PII in photosynthetic organisms [11]. In vitro experiments suggest that a high nitrogen status is sensed by the CrPII protein and CrPII-CrNAGK complex formation is favored, leading to arginine synthesis [10]. However, it remains elusive how these in vitro models accurately mimic cells in vivo. In this work, we report original insights into the NAGK regulation of *Chlamydomonas* under conditions of N-sufficiency or N-limitation.

*Chlamydomonas* efficiently uses ammonium, nitrate, and nitrite as N sources [18,21]. Wild-type cells grown in the media supplemented with ammonium, nitrate, or nitrite with acetate (as a carbon source) showed very similar growth with slightly lower final yields in ammonium-containing medium (Figure 1a and Figure 3a). The highest levels of CrNAGK activity were seen in cells grown in nitrate and nitrite from the early- and mid-log phases. The regulation at the transcriptional level may play a role in higher levels of CrNAGK in cells incubated in nitrate or nitrite compared to that in ammonium (Figure 1b and Figure 3b). Notably, CrPII is also induced by nitrate and nitrite [26], ensuring the possible coordination of two interacted proteins. This result is also consistent with the idea that the PII-dependent and nitrate/nitrite assimilation pathways are interconnecting [20].

We also demonstrated that a certain increase in total Arg accumulation in all N sources was found during the early-log phase of cell growth, especially in nitrite-containing medium (Figure 2b and Figure 3d). In general, an increase in Arg concentrations is expected to limit the accumulation of this amino acid by the inhibition of arginine-sensitive NAGKs [11]. However, there is an additional PII-mediated regulatory mechanism by which high nitrogen availability activates NAGK and thus promotes Arg synthesis [10]. The higher CrNAGK activity in the early-log phase supports the theory that the feedback inhibitory effect of Arg on CrNAGK is alleviated by CrPII in growing cells [11]. As mentioned above, the PII of Chloroplastida has acquired an additional C-terminal extension that acts as a Gln-binding site and makes NAGK activation by PII Gln dependent [9,10,17]. At elevated Gln levels, corresponding to N-rich conditions, CrPII appears to activate CrNAGK (Figure 2c and Figure 3d).

To further study the role of PII-dependent control in the CrNAGK activity of growing cells, we took advantage of the *ami*RNA approach [33]. The two Cr*GLB1*-underexpressing strains that were generated and characterized in this work exhibited a significantly reduced level of PII protein—∼95% less than that in parental strains (Figure 4a)—which is consistent with the low levels of Cr*GLB1* transcripts in both transformants (Figure 4b). The *ami*RNA*GLB1* strains had growth curves that were indistinguishable from the representative growth curve of the WT (Figure S1).

**Figure 7:**
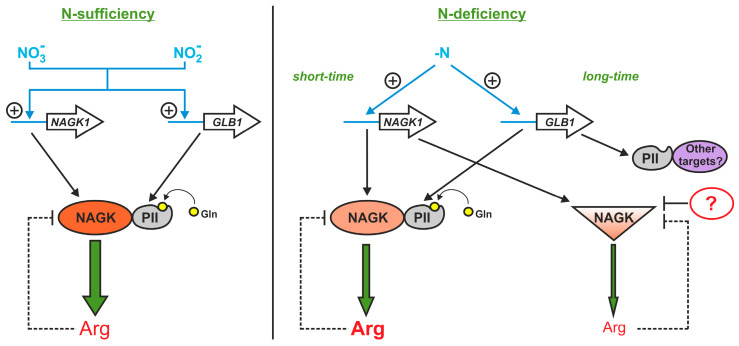
Anticipated model of CrNAGK regulation under N-sufficiency and N-limitation. When N is available, the L-glutamine concentration increases, resulting in PII-NAGK complex formation. Under these conditions, PII alleviates NAGK from Arg feedback inhibition and thereby enhances NAGK activity and Arg production. In addition, nitrate and nitrite upregulate *NAGK1* and *GLB1* gene expression. When cells became N deficient for a short period of time, an increase in NAGK and PII levels appears to be enough to contribute to NAGK-PII complex formation and keep the enzyme active, which in turn results in elevated Arg synthesis. Under N-limitation for a long period of time, NAGK activity decreases and becomes the lowest. This could be achieved by releasing signal protein from the complex with NAGK through a reduction in Gln content and/or PII sequestration by an additional target. The release of PII results in stronger arginine feedback inhibition of NAGK, diminishing energy consumption and flux into arginine. An additional mechanism responsible for negative control of NAGK at the post-translational level is proposed. The positive transcriptional regulation is indicated by (+). The width of the green arrows is indicative of the levels of Arg biosynthesis. Blunted lines denote the negative regulation at the post-translational level.

Compared to the WT, CrNAGK activity was significantly decreased in the *ami*RNA*GLB1* cells from the lag and log phases both in ammonium- and nitrite-containing media (Figure 5a,b). Thus, our results provide primary evidence that the CrPII is a component of CrNAGK regulation in growing cells (Figure 7). Moreover, the Cr*NAGK1* gene was not impaired in the Cr*GLB1*-underexpressing strains (Figure 5c,d), indicating the role of transcription in the regulation of CrNAGK in addition to PII.

Surprisingly, no detectable difference in CrNAGK activity occurred between parental strains and CrPII-transformants in the stationary phase (Figure 5a,b). A possible scenario is that N is depleted in the stationary phase and increased levels of 2-oxoglutarate [13] may interfere with CrPII in the control of CrNAGK [10,11]. However, we cannot rule out the role of the other signals that might arise in this growth phase.

In *Chlamydomonas*, N supply is critical to the maintenance of growth and division [34]. Under N deprivation, Cr*NAGK1* and Cr*GLB1* genes are induced [22,25]. Moreover, in response to -N-shift conditions, CrNAGK activity temporarily increased (Figure 6a,b). A similar transient increase in this enzyme activity following N- deprivation has also been shown in another green alga, *Myrmecia incisa* [17]. Notably, in the case of *amiGLB1* strains, we observed a significant difference in enzyme activity only at 4 h of N deficiency (Figure 6b) Thus, while PII reduction influenced the regulation of CrNAGK in cells subjected to brief N starvation, it appeared not to have a significant effect on the enzyme activity in cells subjected to prolonged N starvation (Figure 7). Since in *Chlamydomonas* the cell density approximately doubles within the first 24 h of N starvation [34], the observed PII-dependent CrNAGK regulation can be used to maintain the finite reservoir of intracellular arginine (Figure 6c). This led to an assumption that another mechanism may be responsible for controlling CrNAGK to adapt to prolonged N limitation.

Although further research into the molecular mechanisms underlying CrNAGK control is required, this study shows that CrPII is only partly responsible for enzyme activity levels in *Chlamydomonas* cells.

## 4. Materials and Methods

### 4.1. Strains and Growth Conditions

The *Chlamydomonas* WT strain 6145c was kindly provided by Prof. Emilio Fernández (University of Córdoba, Spain). The strains CC3491 and CC4533 were obtained from the *Chlamydomonas* Resource Center (University of Minnesota, St. Paul, MN, USA).

Cells were grown in tris-acetate-phosphate (TAP) medium [35] with modified trace elements [36] in a chamber (KBWF 240, Binder GmbH, Tuttlinger, Germany) at 22 °C under continuous illumination by white light (fluence rate of 45 μmol photons/m^2^ s) with continuous agitation (100 rpm). Depending on the nitrogen source, three variants of the TAP medium were used: with either 7.5 mM NH_4_Cl, 4 mM KNO_3_, or 10 mM KNO_2_ as described previously [26,35].

At each harvesting time, the number of cells was recorded by employing a counting chamber. Four hundred cells from each sample were scored for three biological replicates. The number of viable cells was counted microscopically with the use of 0.05% (*v/v*) Evans blue (Dia-M, Moscow, Russia) as previously described [37]. The number of non-viable (stained) and viable (unstained) cells were determined.

### 4.2. Generation of GLB1-Underexpressing Transformants

Screening for *GLB1*-underexpressing transformants was carried out with the plasmid generated previously [33]. The *ami*RNA construct (pChlamiRNA3*GLB1*) or the empty vector [38] was transformed into the cell-wall-deficient CC3491 strain by vortexing with glass beads [39]. The strains were selected on TAP agar containing 10 µg/mL paromomycin (Sigma-Aldrich, Steinheim, Germany) and then screened by Western blotting and RT-qPCR for transformants with reduced abundance of CrPII.

### 4.3. Quantitative Real-Time PCR

The total RNA was isolated with Trizol according to the manufacturer’s instructions (Invitrogen, Waltham, MA, USA). DNA contamination was avoided by treatment of the RNA samples with RNase-Free DNase I (Fermentas, Vilnius, Lithuania). Subsequently, the concentration and purity of total RNA (260/280 nm ratio) were determined using a spectrophotometer (SmartSpec Plus, Bio-Rad Laboratories, CA, USA). Agarose gel electrophoresis (1.2% agarose, w/v) was performed to visualize the integrity of RNA. Reverse transcription was performed with Revert Aid H Minus First Strand cDNA Synthesis Kit according to the manufacturer’s instructions (Thermo Fisher Scientific, No. K-1631, Rockford, IL, USA). Gene expression analysis was carried out by real-time quantitative RT-PCR (RT-qPCR) on a Light Cycler Instrument (CFX96 Real-Time PCR Detection System, Bio-Rad Laboratories, Singapore) using SYBR Green I following a previously reported protocol [22]. The primer pairs used for RT-qPCR were as follows: 5′- GCAGGCGCTCAACATCAACG-3′ and 5′-CATGCCACCAGCAATGACGC-3′ for Cr*NAGK1* (Cre01.g015000_4532), 5′-GGCGTCAAGTTCTTCCGCAT-3′ and 5′-GGTTGGAGGGACCGAACTCA-3′ for Cr*GLB1* (Cre07.g357350_4532) and 5′- CTTCTCGCCCATGACCAC-3′ and 5′-CCCACCAGGTTGTTCTTCAG-3′ for *RACK1* (receptor of activated protein kinase C; Cre06.g278222_4532, formerly termed *CBLP*).

The relative gene expression ratios were normalized with *RACK1* (using the ΔC_T_ and ΔΔC_T_ methods [40]). ΔΔ*C_T_* was used to directly demonstrate the levels of induction and Δ*C_T_* to show relative transcript abundances in selected conditions. Controls without template or reverse transcriptase were always included. The accuracy and reproducibility of the real-time assay were determined by the low variation in C_T_ values across replicates. Values were obtained from at least three biological replicates; each replicate was analyzed three times.

### 4.4. Protein Isolation, SDS-PAGE, and Western Blotting

*Chlamydomonas* cells (4·10^6^ cells/mL in 10 mL) were collected by centrifugation (3000× *g*, 5 min) and resuspended in 0.1 M DTT, 0.1 M Na_2_CO_3_. Then, 0.66 vol of 5% SDS, 30% sucrose was added. Homogenization of the suspensions was achieved by rapid shaking at room temperature for 20 min. The protein concentration was determined by staining with amido black using BSA as a standard [41]. After separation of the proteins by SDS-PAGE on a 12% polyacrylamide gel [42], they were transferred to nitrocellulose membranes (Carl Roth, Karlsruhe, Germany) by semidry blotting (Trans-blot SD, Bio-Rad, Bio-Rad Laboratories, Geylang, Singapore). Blots were blocked in 5% non-fat dry milk in Tris-buffered saline solution with 0.1% Tween 20 prior to incubation in the presence of primary antibodies. The dilution of the primary antibody was 1:5000 anti-PII [22]. As a secondary antibody, the horseradish peroxidase-conjugated anti-rabbit serum (Sigma-Aldrich, No. A054, Steinheim, Germany) was used at a dilution of 1:10,000. The membranes were scanned using Bio-Rad ChemiDocTMMP Imaging System (Bio-Rad, Bio-Rad Laboratories, Singapore).

### 4.5. NAGK Activity Assays

To determine NAGK activity, *Chlamydomonas* strains (2 × 10^6^ cells/mL) grown on ammonium, nitrate, or nitrite were used. Cells were harvested from lag, log, or stationary phase of growth by centrifuging them at 3000× *g* for 10 min, and after resuspending in 200 µL of buffer, pH 7.4 (50 mM Tris-Cl, 0.5 mM EDTA, 1 mM DTT and 0.5 mM benzamidine), they were disrupted by glass beads (0.45 mm diameter) using disintegrator (Minilys, Bertin technologies, Montigny-le-Bretonneux, France) The suspension was centrifuged at 20,000× *g* for 15 min, and the resulting supernatant was used as source of enzyme. All operations were performed at 0 °C. Protein concentration was assessed by the Pierce™ BCA protein assay kit (No 23227, Thermo Fisher Scientific, Rockford, IL, USA).

NAGK activity was measured as previously described [12]. Briefly, freshly prepared protein extracts (0.4 mg) were added to a reaction mixture (400 µL) containing 400 mM NH_2_OH·HCl, 400 mM Tris (base), 20 mM MgCl_2_, and 10 mM ATP. The reaction was started by adding 40 mM N-Acetyl-L-glutamate. After incubation at 37 °C for 1 h, the reaction was stopped by the addition of 400 μL of a solution containing 5% (*w*/*v*) FeCl_3_·6 H_2_O, 8% (*w*/*v*) trichloroacetic acid, and 0.3 M HCl. Blank reactions were performed by omitting N-acetyl-glutamate from the assay. The activity was determined spectrophotometrically following the hydroxamate Fe^3+^ complex formation using a molar extinction coefficient of 456/M cm at 450 nm. One unit of NAGK refers to the amount of enzyme required to catalyze the conversion of 1 mmol of N-acetyl-glutamate/min. The specific activity of CrNAGK was expressed as units per mg of protein. Measurements were performed on at least three separate cultures (biological repeats).

### 4.6. Determination of Glutamine Content

To determine the intracellular Gln levels, the cells (2 × 10^8^ cells/mL) were pelleted (4000× *g*, 5 min) and resuspended in 200 µL of distilled H_2_O for 20 min at 95 °C. Gln content was measured with Glutamine/Glutamate Determination Kit (No. GLN1, Sigma-Aldrich, Steinheim, Germany) according to the manufacturer’s instructions. Briefly, Gln content was determined spectrophotometrically at 340 nm following enzymatic deamination of L-glutamine and dehydrogenation of L-glutamate with conversion of NAD^+^ to NADH [43]. The conversion of NAD^+^ to NADH is proportional to the amount of Glu that is oxidized and, consequently, the amount of Gln converted to Glu in the samples. In addition, endogenous Glu was determined and subtracted from Glu concentration derived by deamination of Gln. The absorbance at 340 nm was measured using a microplate reader CLARIOstar (BMG, Ortenberg, Germany).

### 4.7. Determination of Arginine Content

To determine the intracellular Arg levels, cells (2 × 10^6^ cells/mL) were pelleted (4000× *g*, 5 min) and resuspended in 200 µL of distilled H_2_O for 20 min at 95 °C. Total free Arg was measured as previously described [44] Briefly, 0.1 mL of 0.2% 8-hydroxyquinoline and 0.1 mL of 2 M NaOH were added to the supernatant and the reaction mixture was incubated for 10 min on ice. After addition of 0.1 mL of 19% sodium hypochlorite and vortexing for 30 s, the reaction was stopped by 0.1 mL of 40% urea. The absorbance was estimated at 500 nm. Measurements were performed for at least three biological triplicates.

### 4.8. Statistical Analysis

The values for the quantitative experiments described above were obtained from at least three independent experiments with no fewer than three technical replicates. Data represent the mean ± SE. When necessary, statistical analyses were followed by a Student *t*-test (*p*-value < 0.01 or <0.05) and Pearson’s correlation coefficient *r*.

## Data Availability

The data presented in this study are available on request from the corresponding author.

## Supplementary Materials

The following supporting information can be downloaded at: https://www.mdpi.com/article/10.3390/ijms241612873/s1.
