# Supplementary figures and images for: N-Acetyl-L-glutamate Kinase of Chlamydomonas reinhardtii: In Vivo Regulation by PII Protein and Beyond
Source: Int J Mol Sci. 2023 Aug 17;24(16):12873. doi: 10.3390/ijms241612873 (PMC10454706; doi:10.3390/ijms241612873)

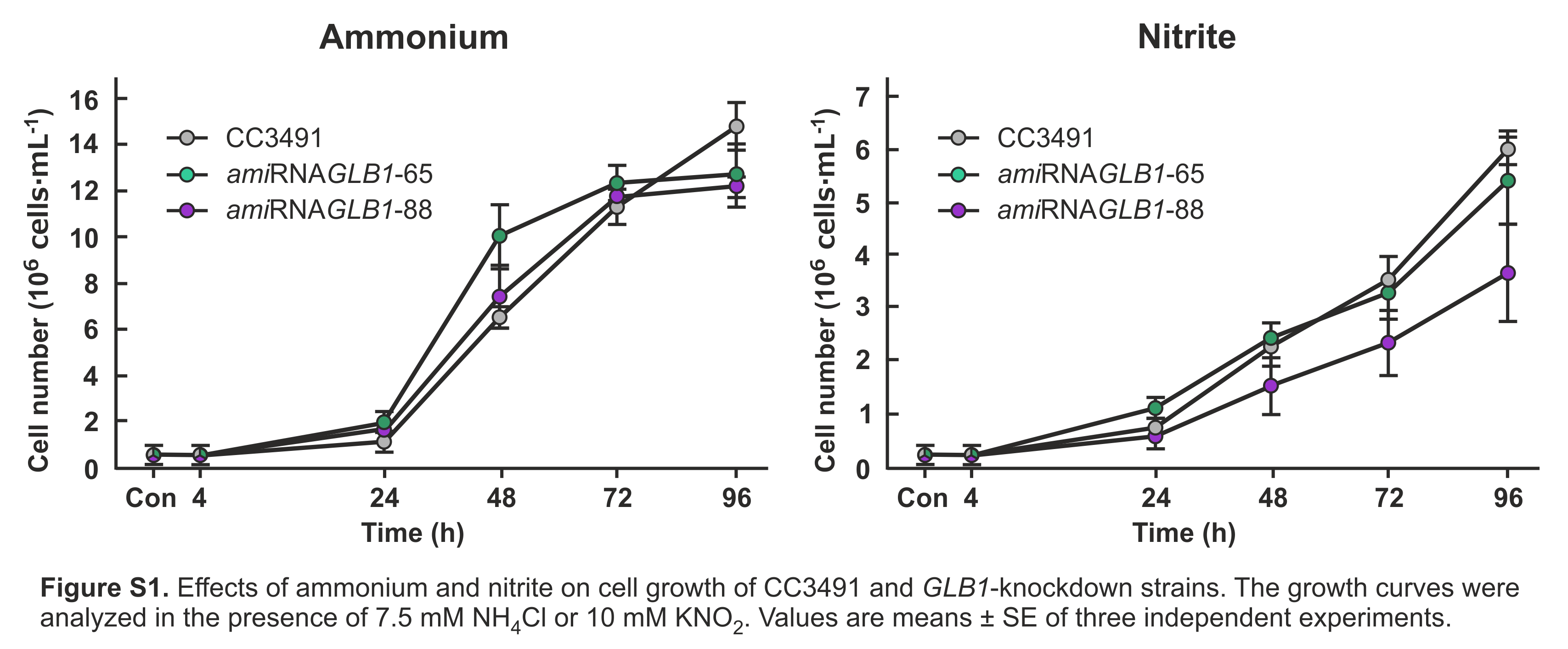

Supplement: Supplementary file 1 [file ijms-24-12873-s001.zip › ijms-2546760-supplementary.tif]
